# Sporozoite neutralizing antibodies elicited in mice and rhesus macaques immunized with *a Plasmodium falciparum* repeat peptide conjugated to meningococcal outer membrane protein complex

**DOI:** 10.3389/fcimb.2012.00146

**Published:** 2012-11-30

**Authors:** Craig Przysiecki, Bob Lucas, Robert Mitchell, Daniel Carapau, Zhiyun Wen, Hui Xu, Xin-Min Wang, Debbie Nahas, Chengwei Wu, Robert Hepler, Elizabeth Ottinger, Jan ter Meulen, David Kaslow, John Shiver, Elizabeth Nardin

**Affiliations:** ^1^Vaccines Research, Merck Research Laboratories, West PointPA, USA; ^2^Division of Medical Parasitology, Department of Microbiology, New York University School of MedicineNew York, NY, USA; ^3^National Center for Advancing Translational Sciences (NCATS), National Institutes of HealthBethesda, MD, USA; ^4^Research Medicine, Merck Research LaboratoriesUpper Gwynedd, PA, USA

**Keywords:** *P. falciparum*, vaccine, sporozoites, peptide-OMPC, mice, rhesus, antibody

## Abstract

Antibodies that neutralize infectivity of malaria sporozoites target the central repeat region of the circumsporozoite (CS) protein, which in *Plasmodium falciparum* is comprised primarily of 30–40 tandem NANP tetramer repeats. We evaluated immunogenicity of an alum-adsorbed (NANP)_6_ peptide conjugated to an outer membrane protein complex (OMPC) derived from *Neisseria meningitidis*, a carrier protein used in a licensed *Haemophilus influenzae* pediatric vaccine. Mice immunized with (NANP)_6_-OMPC adsorbed to Merck's alum adjuvant (MAA), with or without Iscomatrix® as co-adjuvant, developed high levels of anti-repeat peptide antibody that inhibited *in vitro* invasion of human hepatoma cells by transgenic *P. berghei* sporozoites that express *P. falciparum* CS repeats (PfPb). Inhibition of sporozoite invasion *in vitro* correlated with *in vivo* resistance to challenge by the bites of PfPb-infected mosquitoes. Challenged mice had >90% reduction of hepatic stage parasites as measured by real-time PCR, and either sterile immunity, i.e., no detectable blood stage parasites, or delayed prepatent periods which indicate neutralization of a majority, but not all, sporozoites. Rhesus macaques immunized with two doses of (NANP)_6_-OMPC/MAA formulated with Iscomatrix® developed anti-repeat antibodies that persisted for ~2 years. A third dose of (NANP)_6_-OMPC/MAA+ Iscomatrix® at that time elicited strong anamnestic antibody responses. Rhesus macaque immune sera obtained post second and third dose of vaccine displayed high levels of sporozoite neutralizing activity *in vitro* that correlated with presence of high anti-repeat antibody titers. These preclinical studies in mice of different MHC haplotypes and a non-human primate support use of CS peptide-OMPC conjugates as a highly immunogenic platform to evaluate CS protective epitopes. Potential pre-erythrocytic vaccines can be combined with sexual blood stage vaccines as a multi-antigen malaria vaccine to block invasion and transmission of *Plasmodium* parasites.

## Introduction

Malaria caused by the parasite *Plasmodium falciparum* is considered one of the most prevalent and deadliest of diseases. The complexity of the *Plasmodium* life cycle, which involves multiple parasite stages in the mosquito vector and in the mammalian host, necessitates a multipronged control effort, ideally involving a combination of chemotherapy, vector control, and vaccines. Despite the fact that 40% of the world's population is at risk of malaria, with 300–500 million cases and 1 million deaths each year, there is no licensed malaria vaccine available.

One of the lead vaccine candidates in clinical trials is the circumsporozoite (CS) protein which is a major surface protein of the infective sporozoite. A Phase III trial is in progress of a CS-based pediatric malaria vaccine RTS,S which can protect 35–40% of African infants against clinical disease (Agnandji et al., 2011). Immunization with RTS,S in a potent adjuvant formulation elicited sterile immunity in 30–40% of malaria-naïve volunteers, however, only transient protection against infection was obtained in African adults (Bojang et al., 2001; Kester et al., 2009). Protection correlated with high levels of anti-repeat antibodies and CS-specific CD4+ T cells (Kester et al., 2009; Olotu et al., 2010, 2011). While these studies support the feasibility of a CS-based subunit vaccine, efforts continue to increase immunogenicity and efficacy of malaria vaccines using new adjuvant and delivery platforms.

The first trial of a malaria peptide vaccine directly targeting the CS repeats was the peptide-conjugate vaccine using tetanus toxoid (TT) as carrier protein, (NANP)_3_-TT, which elicited anti-repeat antibodies that protected a small number of immunized volunteers challenged by exposure to the bites of *P. falciparum*-infected mosquitoes (Herrington et al., 1987). These studies provided proof-of-principle that a CS repeat peptide vaccine could elicit protective immunity in humans, however, the magnitude of anti-repeat antibody responses in this first clinical trial were suboptimal. The humoral response was dose dependent and a major limitation of the vaccine was the low density of peptide that could be conjugated to the TT carrier. The outer membrane protein complex (OMPC) of *Neisseria meningitidis* is an attractive carrier protein as it provides high density peptide conjugation. OMPC has a clinical track record as a carrier for polysaccharides in a pediatric *Haemophilis influenzae* type b (Hib) vaccine, PedvaxHIB® (Merck), used safely in millions of infants world-wide (Zhou et al., 2002). The use of a carrier with prior applications in commercial pediatric vaccines would be particularly attractive for a malaria vaccine, as infants suffer the majority of the one million malaria deaths in Africa, and scale-up production, safety, and acceptability have been previously established. In previous studies, we have shown that a conjugate of OMPC to a *P. falciparum* gamete/ookinete protein, Pfs25, elicited high titers of transmission blocking antibodies in mice and rhesus macaques that reduced mosquito infection (Wu et al., 2006).

In the initial assessment of OMPC as carrier for *P. falciparum* CS repeats, synthetic peptide containing variable numbers of the *P. falciparum* NANP tetramer were conjugated to OMPC and tested with various adjuvants for immunogenicity in mice and rhesus macaques. In inbred strains of mice, (NANP)_6_-OMPC/Merck alum adjuvant (MAA) immunization elicited high levels of anti-repeat antibodies that neutralized sporozoite infectivity *in vitro* and *in vivo*. In rhesus macaques, (NANP)_6_-OMPC/MAA formulated with Iscomatrix® elicited anti-repeat antibodies that persisted for two years following a prime and boost immunization and strong anamnestic antibody responses were obtained following a second boost. The sporozoite neutralizing activity in the rhesus macaque sera correlated with levels of anti-repeat antibodies and neutralizing activity could be inhibited by presence of repeat peptide in a peptide competition assay. These promising studies in inbred and outbred animals support efforts to develop OMPC conjugates containing multiple malaria antigens to elicit immune responses that effectively block transmission of the parasite to the mammalian host as well as to the mosquito vector.

## Materials and methods

### Synthetic peptides

The *P. falciparum* CS repeat tetramers, (NANP)_3_ and (NANP)_6_, were synthesized as bromoacetylated peptides with the latter peptide also synthesized having the bromoacetyl group at the C-terminus. A spacer 6-aminohexanoic acid (Aha) was incorporated between the repeats and BrAc. The non-bromoacetylated containing terminus of the peptide was blocked with an N-acetyl or carboxamide group to give final constructs:

BrAcAha(NANP)_3_NH_2_: BrAc-Aha-NANPNANPNANP-NH_2_

BrAcAha(NANP)_6_NH_2_:   BrAc-Aha-NANPNANPNANPNAN PNANPNANP-NH_2_

Ac(NANP)_6_LysAhaBrAc-NH_2_:   Ac-NANPNANPNANPNANP NANPNANP-Lys (Aha-BrAc)-NH_2_

Peptides were cleaved from the resin with a mixture of 95% TFA, 2.5% water, and 2.5% triisopropylsilane. The crude peptide product was lyophilized to dryness, re-suspended in 50% acetic acid and water (v:v), and purified by preparative RP-HPLC. Fractions were analyzed by LC/MS HPLC. Fractions with correct mass and >95% homogeneity by peak area were pooled and lyophilized to dryness.

### Conjugation of CS repeat peptides to OMPC

OMPC was obtained from Merck Manufacturing Division (West Point, PA). A portion of OMPC surface amines were aseptically thiolated using N-acetylhomocysteinethiolactone (Aldrich, St. Louis, MO.) in N_2_-sparged borate buffered saline (25 mM sodium borate, pH 8.5, 0.15 M NaCl), as previously described (Wu et al., 2006), with the final thiolated OMPC re-suspended in N_2_-sparged 25 mM sodium borate, pH 8.5, 0.15 M NaCl. Free thiol content was determined by Ellman assay and measured thiol content was between 0.2 and 0.3 μmol/mL. Peptides were re-suspended in N_2_-sparged borate buffered saline (25 mM borate, 0.15 M sodium chloride, pH 8.5) at 5 mg/mL and 0.22 μm filtered. Peptides were mixed with the thiolated OMPC solution at a final 1.5-fold molar excess of peptide BrAc to total OMPC thiol. The reaction was protected from light and incubated at ambient temperature for ~22 h. Residual free OMPC thiol groups were quenched with a 5-fold molar excess of N-ethylmaleimide for 4 h at ambient temperature. Quenched conjugates were dialyzed exhaustively against borate buffered saline dialysis buffer in Spectra-Por 100,000 Da MWCO dialysis units (Spectrum Labs, Rancho Dominguez, CA). Any aggregated material in the dialyzed product was removed by centrifugation at 2,280 × g for 5 min at 4°C.

### Conjugate analysis

Total protein content was determined by a modified Lowry assay (Markwell et al., 1978) and aliquots of conjugate and control OMPC (thiol activated OMPC, N-ethylmalemide quenched, and dialyzed) were analyzed by quantitative amino acid analysis (AAA). Peptide to OMPC molar ratios were determined from quantitation of the unique residue S-carboxymethylhomocysteine (SCMHC) which was released upon acid hydrolysis of the nascent peptide-OMPC bond (Nahas et al., 2008). The OMPC-specific concentration was determined from hydrolysis-stable residues which were absent from the peptide sequence and thus unique to OMPC protein. Assuming 1 mol of peptide for every mol SCMHC, the ratio of SCMHC/OMPC was thus equivalent to the peptide/OMPC content. The mass loading of peptide could be calculated from this ratio using the peptide molecular weight and an average OMPC mass of 40,000,000 Da. The covalent nature of the conjugation was qualitatively confirmed by SDS-PAGE analysis using 4–12% Bis-Tris NuPAGE gels (Invitrogen, Carlsbad, CA) where an upward shift in mobility was observed for the Coomassie-stained conjugate bands relative to control activated and quenched OMPC (data not shown).

### Adjuvant formulation

Conjugates were adsorbed to Merck's amorphous aluminum hydroxyphosphate sulfate adjuvant (MAA) (Merck Manufacturing Division, West Point, PA) to give a final aluminum concentration of 0.45 mg/ml. The Al^3+^/dose were 0.045 mg for mice and 0.225 mg for rhesus macaques. In some experiments, a saponin-based adjuvant, Iscomatrix® (CSL, Melbourne, VIC, Australia), was added to the MAA-adsorbed peptide conjugate to give a final concentration of 0.025 mg Isco units/mL for mouse preparation (2.5 μg/dose) and 0.1 mg Isco units /mL for the rhesus macaque preparation (50 mcg/dose).

### Murine immunogenicity studies

All murine studies were approved by IACUC at New York University School of Medicine and Merck Research Laboratories (West Point, PA). Female Balb/c mice (Taconic Farms) or C57Bl/6 mice (Jackson Labs, Me), 6–8 weeks of age (*n* = 5–10/group) were given three 4 μg doses of conjugated peptide on days 0, 14, and 28 by hind leg intramuscular (IM) injection. Three constructs were tested that differed in number of repeats and peptide orientations: conjugation to N-terminus of (NANP)_3_ or (NANP)_6_ peptide [OMPC-Aha(NANP)_3_NH_2_/MAA and OMPC-Aha (NANP)_6_/MAA, respectively], and C-terminal conjugation of (NANP)_6_-OMPC/MAA. The (NANP)_6_-OMPC/MAA C-terminal conjugation was evaluated with and without the addition of Iscomatrix® as co-adjuvant. Control mice were immunized with activated/quenched but non-conjugated OMPC adsorbed to MAA. In an additional experiment, mice received two doses of OMPC/MAA prior to immunization with single dose of (NANP)_6_-OMPC/MAA. Blood samples were obtained for serum prior to each immunization and at various time points post second dose to measure antibody persistence. Serum was stored at −20°C until assayed.

### Rhesus macaques immunogenicity studies

Adult *Macaca mulatta* rhesus macaques of Indian origin, 6 years of age at the start of the study, were housed at an AAALAC approved facility at New Iberia Research Center (NIRC), New Iberia, LA. Rhesus macaque studies were approved by IACUC at NIRC and Merck Research Laboratories. Groups of three adult rhesus macaques were given three 4 μg doses of (NANP)_6_-OMPC/MAA on days 0, 70, and 732 by deltoid IM injection. The MAA adsorbed peptide conjugate was tested with and without Iscomatrix® as co-adjuvant. Blood was drawn intravenously at days 1, 35, 69, 84, 140, 200, 300, 365, 732, and 760. Sera was generated from the blood samples using serum separators and stored at −70°C.

### Serologic assays

Enzyme-linked immunoadsorbent assay (ELISA) was carried out to measure anti-repeat antibodies using as coating antigen either (NANP)_3_ tetrabranched peptide (Nardin et al., 1995) or R32, an *E. coli* expressed recombinant protein containing 32 tetramer repeats (Young et al., 1985). Briefly, 96-well plates (Nunc Immuno Plate, MaxiSorp or Immunolon 2HB) were coated with 100 ng/well of recombinant R32 protein or with 1 μg/ml tetrabranched (NANP)_3_ peptide. Murine or rhesus macaque sera were tested at 2-fold dilutions starting at 1:80 dilution. MAB 2A10, specific for *P. falciparum* CS repeats, was titrated on each plate as a positive control. Plates were washed using PBS with 0.05% Tween20 prior to addition of species specific peroxidase-labeled anti-IgG antibody (Sigma-Aldrich, St. Louis, MO; or Kirkegaard, Perry Laboratories, Gaithersburg, MD). Plates were subsequently washed after incubation using PBS/0.05% Tween20. Color development was accomplished using ABTS 1:1 (Fisher) and plates read at wavelength A_405 nm_. Antibody endpoints were determined based on a cut-off OD greater than three times OD in BSA-coated wells. The ug/ml of anti-repeat antibody was determined by linear regression based on titration of MAB 2A10.

Indirect immunofluorescence (IFA) was carried out using two-fold dilutions of pooled mouse sera, or individual rhesus macaques sera incubated on multiwell slides containing air-dried *P. falciparum* sporozoites (Othoro et al., 2009). Following washing, slides were reacted with species-specific FITC-labeled anti-Ig antibodies (Kirkegaard, Perry Laboratories; Gaithersburg, MD). Slides were coded and the endpoint titer was defined as the highest sera dilution giving sporozoites with unequivocal positive fluorescence.

The circumsporozoite precipitin (CSP) reaction is a terminal precipitin that forms on viable sporozoites incubated in immune serum due to antibody cross-linking of the surface CS protein (Vanderberg et al., 1969; Cochrane et al., 1976). CSP reactions were carried out using PfPb sporozoites, a transgenic *P. berghei* strain that expresses *P. falciparum* CS repeats (Persson et al., 2002). Equal volumes of sporozoites (1 × 10^6^/ml) and 2-fold dilutions of immune sera were incubated for 45 min at 37°C. The endpoint titer was defined as the last serum dilution giving 2+ CSP reactions in a total of 20 sporozoites when examined by phase-contrast microscopy.

### Transgenic sporozoite neutralization assay (TSNA)

The presence of functional inhibitory antibodies was determined *in vitro* using PfPb sporozoites, transgenic *P. berghei* parasites expressing *P. falciparum* repeats (Kumar et al., 2004; Othoro et al., 2009). PfPb sporozoites (2 × 10^4^) were incubated with serial dilutions of murine or rhesus macaque sera for 40 min on ice prior to addition to 24 well plates containing confluent HepG2 cells. Controls included sporozoites incubated with 25 μg/ml of MAB 2A10, specific for *P. falciparum* CS repeats or MAB 3D11 specific for *P. berghei* CS repeats, or with medium only. Plates were cultured for 48 h at 37°C and 5% CO_2_, harvested and total RNA extracted and reverse transcribed. Parasite levels were quantitated by real-time qPCR using primers for parasite 18S ribosomal RNA, as previously described (Bruna-Romero et al., 2001; Othoro et al., 2009).

### Challenge by exposure to bites of PfPb-infected mosquitoes

Immunized and naïve C57Bl/6 mice were anesthetized and challenged by exposure for 15 min to the bites of 10–20 *Anopheles stephansi* mosquitoes infected with PfPb transgenic rodent parasites expressing the *P. falciparum* CS repeats. Forty hours post challenge, mice were sacrificed, livers removed, and RNA extracted for measurement of parasite burden by qPCR, as described for TSNA. The remaining mice where followed by microscopy using daily Giemsa-stained blood smears to determine presence of blood stage parasites. Mice that remained blood stage parasite negative for 14 days post challenge were considered to have sterile immunity.

## Results

### Immunogenicity of (NANP)_6_-OMPC conjugate in inbred strains of mice

Initial studies carried out in BALB/c mice established the number of repeats and peptide orientation required for optimal immunogenicity of the peptide-OMPC conjugates. A peptide-OMPC conjugate containing six NANP repeats, (NANP)_6_-OMPC, was found to elicit higher anti-repeat antibody titers than the conjugate containing three NANP repeats, (NANP)_3_-OMPC. Peptide orientation did not affect immunogenicity, as no difference was observed between N- or C-terminal conjugation of the (NANP)_6_ peptide to OMPC (data not shown). All subsequent studies were therefore carried out using an (NANP)_6_-OMPC conjugate.

**Figure 1:**
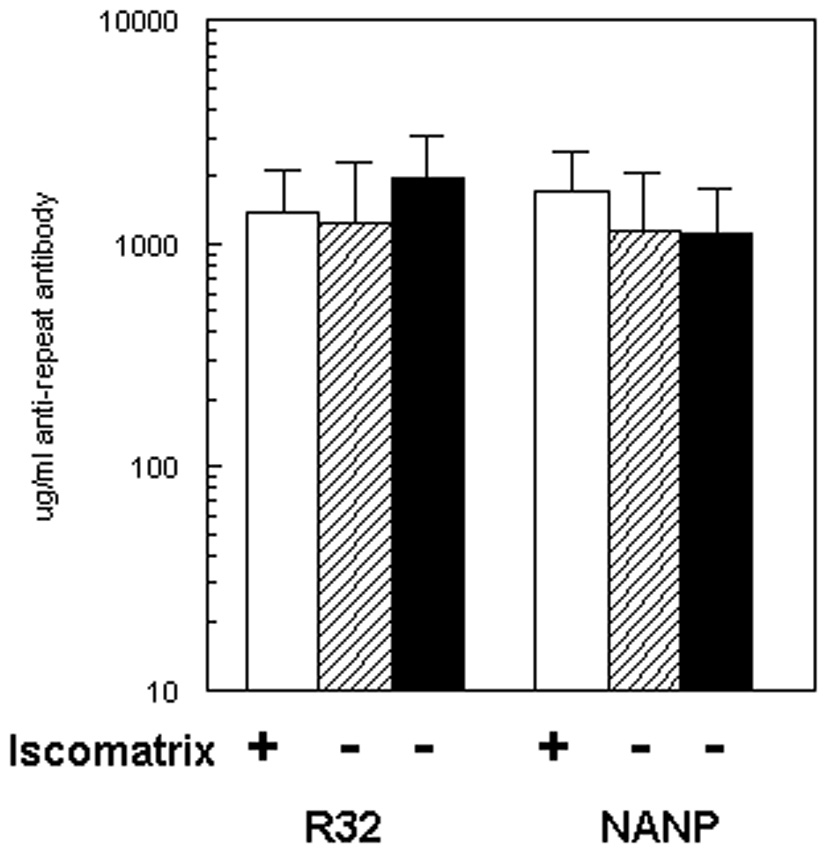
**Immunogenicity of (NANP)_6_-OMPC in BALB/c.** Mice (*n* = 10/group) were immunized with three doses of (NANP)_6_-OMPC/MAA with (open bars) or without (hatched bars) Iscomatrix®. ELISA used either recombinant protein R32 (left panel), or (NANP)_3_ peptide (right panel). Antibody levels obtained with freshly prepared (NANP)_6_-OMPC/MAA (hatched bars) were compared with peptide-conjugate stored for 2 years at 4°C (black bars).

Balb/c mice were immunized on days 0, 14, and 28 by IM injection of 4 μg (NANP)_6_-OMPC (total peptide in conjugate) adsorbed to MAA (Merck amorphous aluminum hydroxphosphate sulfate), with or without Iscomatrix® as co-adjuvant. All conjugates were dosed based on the specific peptide content as measured by quantitative AAA. Following the third dose of (NANP)_6_-OMPC/MAA, peak anti-repeat antibody concentrations of ~1,200 μg/ml were measured by ELISA (Figure 1). The magnitude of the antibody response was comparable using either (NANP)_3_ peptide or recombinant R32 protein coated plates in the ELISA.

Addition of the Iscomatrix® co-adjuvant to the MAA formulation did not significantly increase immunogenicity (Figure 1, open bar). In the second experiment, BALB/c mice gave comparable results when immunized with the same preparation of peptide conjugate which had been stored at 4°C for ~2 years (Figure 1, black bars). The μg/ml of repeat specific antibody in Experiment I was 1,130 μg/ml and in Experiment II 1,110 μg/ml, corresponded to geometric mean endpoint titers (GMT) of 359,480 and 551,090, respectively, when measured against (NANP)_3_ peptide. All subsequent murine studies were carried out using the (NANP)_6_-OMPC/MAA formulation without Iscomatrix®.

**Figure 2:**
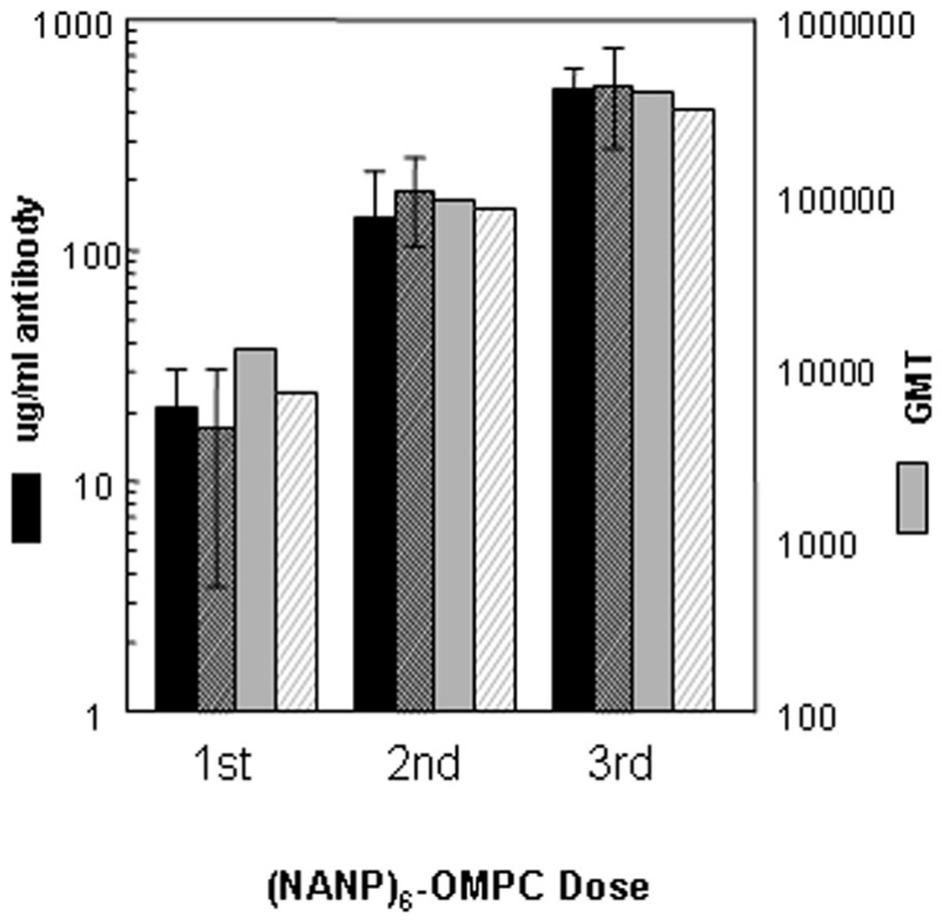
**Immunogenicity of (NANP)_6_-OMPC/MAA in C57Bl/6 mice.** Kinetics of anti-repeat antibody response shown for two experiments (solid bars Experiment I, hatched bars Experiment II). Antibody concentration is shown as μg/ml (left axis) or geometric mean titers (GMT) (right axis), as measured in ELISA using either R32 (black bars) or (NANP)_3_ peptide (gray bars) as coating antigen.

Humoral immune responses in a second inbred strain, C57Bl/6, demonstrated that the high level of anti-repeat antibody induced by (NANP)_6_-OMPC/MAA was not strain dependent and was highly reproducible (Figure 2). In two independent experiments in C57Bl/6 mice, 100% seroconversion was obtained following a single dose of (NANP)_6_-OMPC /MAA, with 17–21 μg/ml of anti-repeat antibody (black solid bars vs. black hatched bars), corresponding to 12,607 vs. 6,967 GMT when expressed as endpoint titers (gray solid bars vs. gray hatched bars). A booster injection on day 14 elicited a 6–12-fold increase in the anti-repeat titers. Peak antibody responses were obtained following the third dose, with an additional 2–5-fold increase to 502–516 μg/ml of anti-repeat antibody, corresponding to GMT 376,405 and 305,736 in Experiment 1 and 2, respectively. The antibodies elicited by (NANP)_6_-OMPC reacted with native CS on sporozoites. The sera from mice immunized with either fresh or stored peptide-conjugate reacted with air-dried *P. falciparum* sporozoites with ~10^4^ IFA titers. The immune sera also elicited high CSP titers (1:125) when incubated with viable PfPb sporozoites expressing *P. falciparum* CS repeats, indicating that the antibodies elicited by the peptide conjugates effectively cross-linked the surface CS protein on viable sporozoites.

OMPC is a carrier for bacterial capsular polysaccharide in a *Haemophilus influenzae* type b pediatric vaccine (PedvaxHIB®), which is widely used to prevent childhood bacterial meningitis. Therefore, a proportion of infants might have antibody to the OMPC carrier that could positively or negatively modulate the response to (NANP)_6_-OMPC vaccine. To test whether anti-OMPC antibodies modulate the anti-repeat response, mice were primed with two doses of OMPC (anti-OMPC GMT 5,120) followed by a single dose of (NANP)_6_-OMPC/MAA.

**Figure 3:**
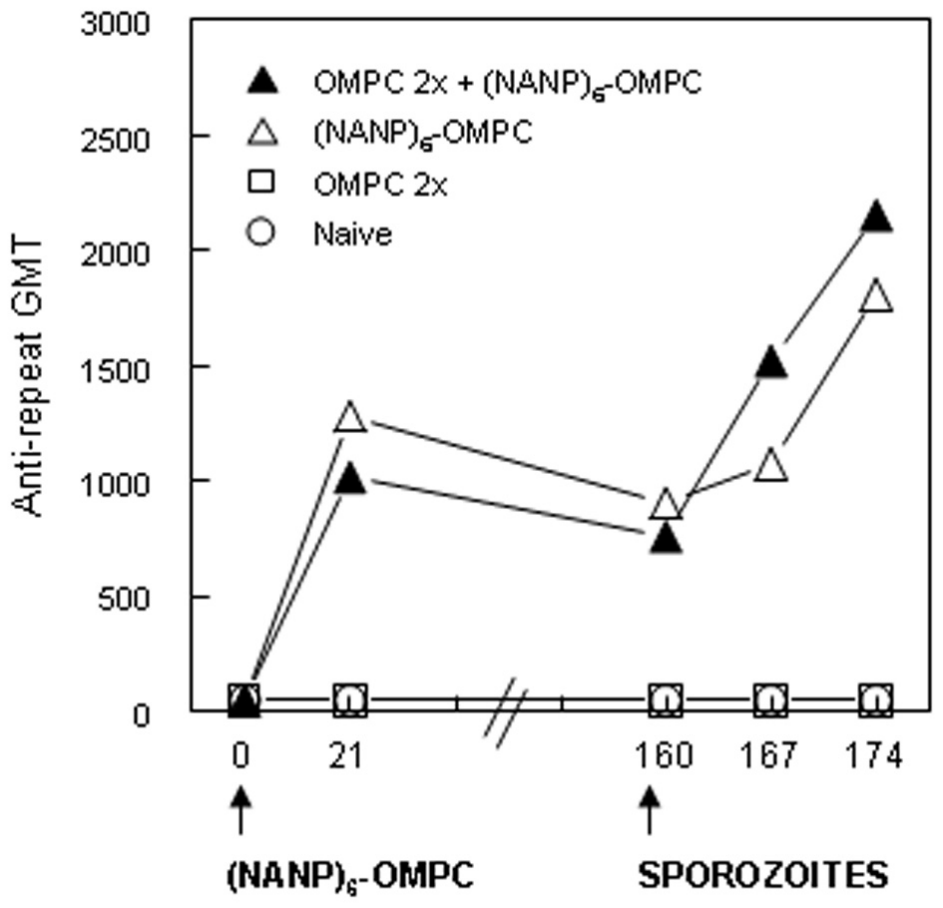
**Immunization of mice with pre-existing anti-OMPC antibody.** A single dose of (NANP)_6−_OMPC/MAA was given to naïve C57Bl/6 (open triangle) or to mice previously immunized with two doses of OMPC/MAA with anti-OMPC GMT 5120 (data not shown) at time of immunization (closed triangle). All groups were exposed to the bites of irradiated PfPb infected mosquitoes on day 160.

The kinetics and magnitude of the anti-repeat antibody in OMPC-immunized mice were comparable to responses elicited when (NANP)_6_-OMPC was used to immunize mice without pre-existing anti-OMPC antibodies (Figure 3). The pre-existing anti-OMPC antibody also did not alter the persistence of the anti-repeat antibody response, with GMT of 761 and 905 at 160 days post the single dose of (NANP)_6_-OMPC/MAA in mice with or without pre-existing anti-OMPC antibodies, respectively. Importantly, the development of malaria-specific memory B cells was also not affected, as exposure to the bites of PfPb infected mosquitoes at day 160 increased anti-repeat antibody responses in both the OMPC-primed and non-primed mice. These findings suggest that the potential presence of anti-OMPC antibodies will not inhibit response to a malaria peptide-OMPC conjugate. In addition, the findings also suggest that the vaccine induced anti-repeat antibody response can be boosted by exposure to the bites of malaria-infected mosquitoes, providing the potential for continued boosters in malaria endemic areas.

### Sporozoite neutralizing antibody in sera of (NANP)_6_-OMPC immunized mice

**Figure 4:**
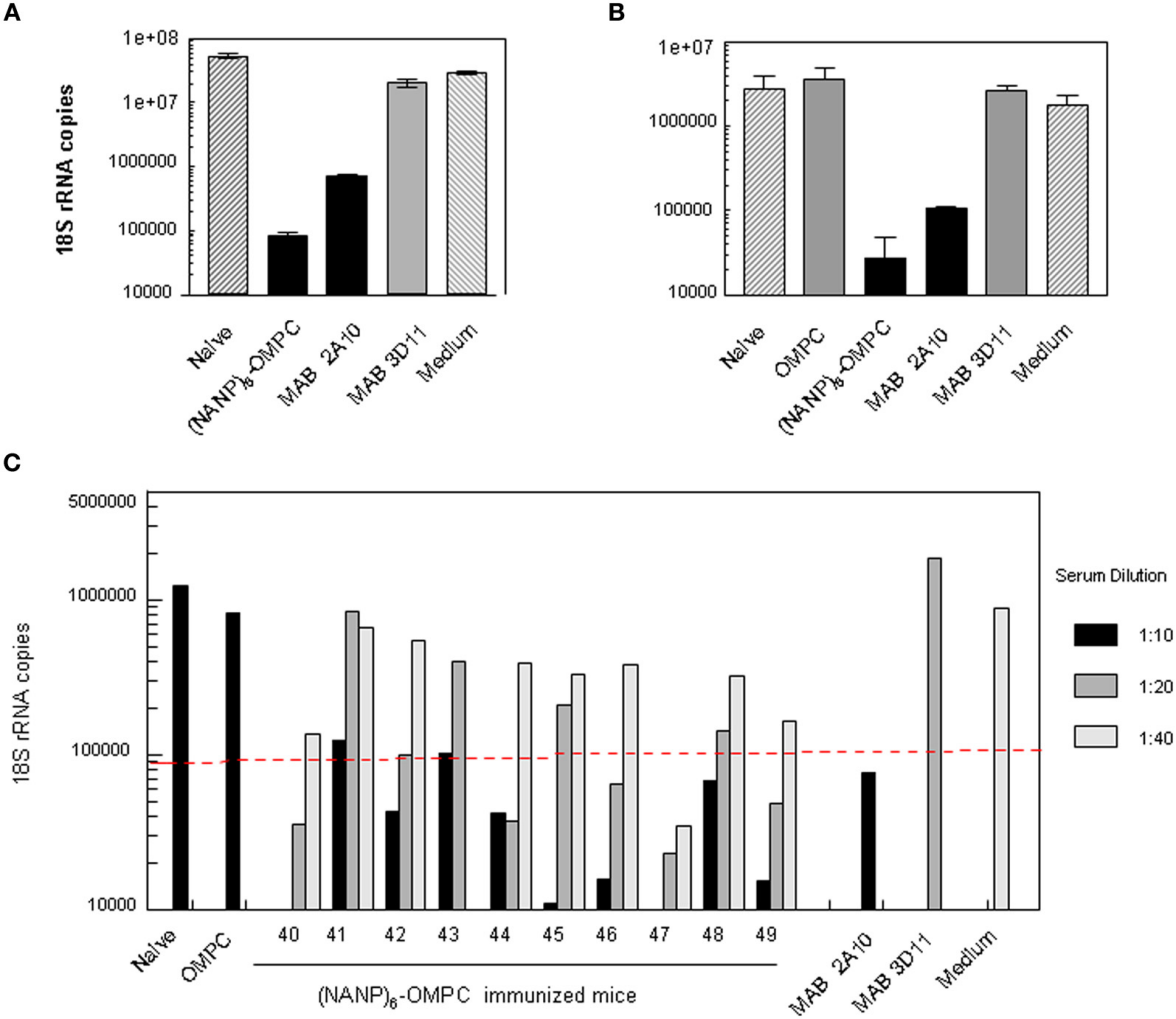
**Sporozoite meutralizing activity in murine immune serum.** Sera from **(A)** Balb/c or **(B)** C57Bl /6 mice immunized with three doses of (NANP)_6_-OMPC/MAA were tested in TSNA. PfPb sporozoites, that express *P. falciparum* CS repeats, were incubated with immune or control sera (1:5 dilution) prior to addition to HepG2 cells. Levels of parasites in HepG2 cell extracts collected after 48 h of culture were measured by qPCR. Results are shown as mean number of parasite 18S rRNA copies in cell extracts, based on an 18S rRNA plasmid standard. **(C)** Individual sera of 10 C57Bl/6 mice were tested in TSNA at dilutions of 1:10–1:40. Dotted line indicates 90% inhibition level.

The high titers of anti-repeat antibody elicited by immunization with (NANP)_6_-OMPC/MAA were found to be functional when tested in an *in vitro* sporozoite neutralizing assay using transgenic *P. berghei* sporozoites expressing *P. falciparum* CS repeats (PfPb) (Kumar et al., 2004). When PfPb sporozoites were pre-incubated with serum from (NANP)_6_-OMPC/MAA immunized BALB/c mice prior to addition to HepG2 cell cultures, the number of intracellular parasites detected after 48 h culture was reduced by ≥90%, when compared to cultures with sporozoites incubated in medium without antibody (Figure 4A). Similar levels of sporozoite neutralizing activity were detected in the serum of C57Bl/6 mice immunized with (NANP)_6_-OMPC/MAA (Figure 4B). The level of inhibition observed with immune serum of BALB/c and C57Bl/6 mice was comparable to that obtained when PfPb sporozoites were incubated with 25 μg/ml of MAB 2A10 specific for *P. falciparum* CS repeats. Neutralizing activity was specific for *P. falciparum* CS repeats, as MAB 3D11 specific for *P. berghei* CS repeats did not inhibit PfPb sporozoite invasion. Control serum from mice immunized with OMPC/MAA only and serum of naïve mice were not inhibitory.

Inhibition was dose-dependent, and lower levels of parasite inhibition were detected at higher serum dilutions (Figure 4C). Sera of 8/10 immunized C57Bl/6 mice gave ≥90% inhibition of liver stage parasites at a 1:10 dilution, while sera of 5/10 immune mice had neutralizing activity >90% at 1:20 dilution. One of nine immune sera tested at 1:40 dilution gave >90% inhibition. Control MAB 3D11 and serum of mice immunized with OMPC/MAA or naïve mice were not inhibitory at lowest dilution tested (1:10).

### Sporozoite neutralizing antibody *in vivo*

To determine if sporozoite neutralization *in vitro* correlated with *in vivo* resistance to infection, the 10 (NANP)_6_-OMPC/MAA immunized mice, along with similar number of OMPC/MAA immunized control and naïve mice, were challenged by exposure to the bites of PfPb-infected mosquitoes. Half of the mice were sacrificed at 40 h post challenge to measure parasite levels in the liver, while the other half were monitored for development of blood stage parasites using daily blood smears.

**Figure 5:**
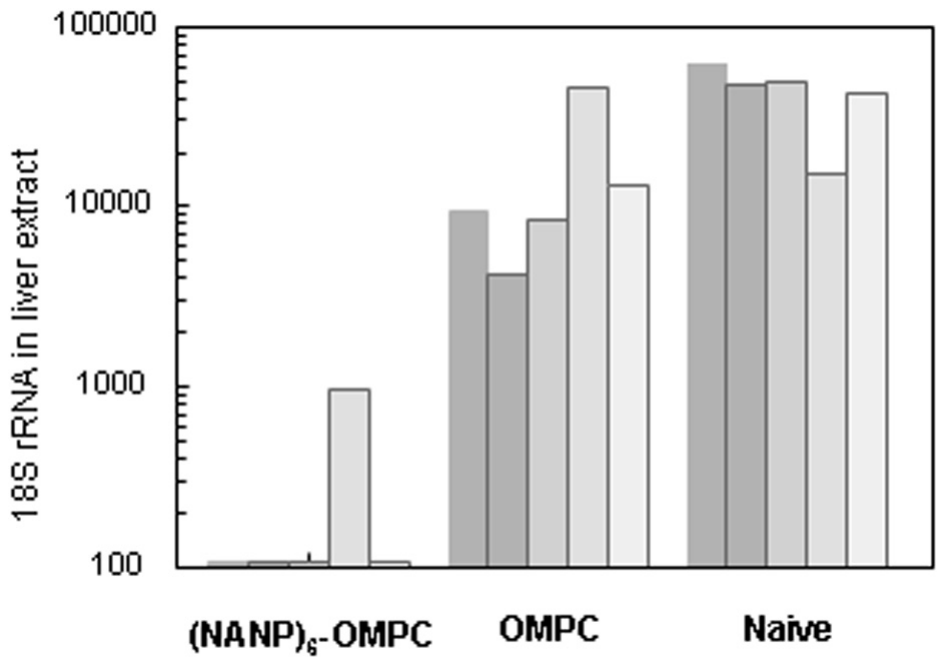
**Parasite levels in murine liver following challenge by bites of infected mosquitoes.** Parasite 18 S rRNA copy numbers in liver extract obtained at 40 h post challenge by bites of 5–15 PfPb-infected mosquitoes was determined by qPCR. Results are shown for five mice in each group.

Reduction of >90% of liver stage parasites relative to naïve mice was detected in 5/5 challenged mice when liver extracts were assayed by qPCR (Figure 5). In 4/5 of the (NANP)_6_-OMPC immunized mice, the number of rRNA copies was below the level of detection by qPCR (<100 18S rRNA copies). The parasite burden in the liver of mice immunized with OMPC only was lower than in naïve mice, suggesting potential non-specific innate immune responses were elicited by OMPC, which is a known TLR2 agonist (Latz et al., 2004) and/or the MAA adjuvant.

The *in vitro* sporozoite neutralization correlated with *in vivo* resistance to sporozoite challenge (Table 1). The remaining five immunized mice in the cohort challenged by bites of PfPb-infected mosquitoes were monitored for development of parasitemia by microscopic examination of daily Giemsa-stained blood smears. No patent blood stage infection was detected in 4/5 immunized mice followed for 16 days post challenge, indicating sterile immunity was elicited by immunization with (NANP)_6_-OMPC. The single (NANP)_6_-OMPC immunized mouse that developed parasitemia had a delayed prepatent period of 7 days, which was significantly prolonged compared to the five naïve mice which all became patent 3–4 days post challenge. The sterile protection in these mice correlate with the low levels of parasite rRNA detected in liver at 48 h post challenge in the qPCR measurements carried out in the parallel cohort (Figure 5).

**Table 1:** **(NANP)6-OMPC immunized mice challenged by exposure to malaria-infected mosquitoes**^***a***^.

**Experiment**	**Group**	**# Pos/total**	**Protected%**	**PPP**^***b***^	**(Days)**
I	(NANP)_6_-OMPC	1+/5	80	7.0	–
	OMPC	5+/5	0	5.0	(4–6)
	Naive	5+/5	0	3.6	(3–4)
II	(NANP)_6_-OMPC	3+/5	40	6.7	(6–8)
	OMPC	5+/5	0	4.0	(3–5)
	Naive	5+/5	0	3.8	(3–4)

a Naïve or immunized C57Bl/6 mice were challenged by exposure to bites of 18 mosquitoes infected with PfPb parasites expressing P. falciparum CS repeats.

b Prepatent period (PPP) is the first day blood stage parasites were detected by microscopy in Giemsa stained daily blood smears.

The development of protective immune responses in (NANP)_6_-OMPC immunized mice was confirmed in a second experiment, in which 2/5 of (NANP)_6_-OMPC immunized mice remained negative after sporozoite challenge (Table 1). The prepatent period in the immunized mice that developed blood stage infection was 6.7 days, vs. 4.0 days for OMPC immunized mice and 3.8 days for naive controls. The prolonged prepatent period in the (NANP)_6_-OMPC immunized mice that developed blood stage infection in Experiments 1 and 2, indicate that the majority, but not all, of the infective sporozoites injected by the mosquito were neutralized *in vivo.* Previous experiments using intravenous injection of know numbers of parasites have shown that elimination of >90% of sporozoites is required to obtain a one day delay in developing a patent blood stage infection.

### Immunogenicity of (NANP)_6_-OMPC conjugates in rhesus macaques

**Figure 6:**
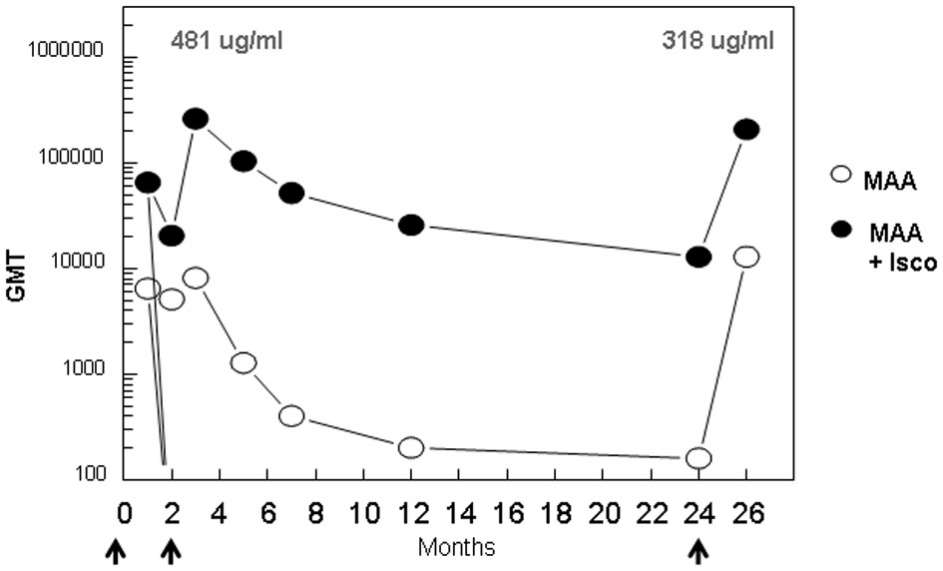
**Kinetics of anti-repeat antibody response in rhesus macaques.** Groups of three adult rhesus macaques were immunized with (NANP)_6_-OMPC/MAA with or without Iscomatrix® on days 0, 70, and 732 (arrows). Sera were collected at indicated time points and results are shown as ELISA GMT using (NANP)_3_ peptide coated plates. The concentration of repeat specific antibody (μg/ml) is shown for peak responses post 1st and 2nd booster immunization.

The kinetics and fine specificity of antibody responses in an outbred non-human primate population, rhesus macaques, was assessed following IM immunization with (NANP)_6_-OMPC/MAA formulated with or without Iscomatrix® co-adjuvant. In contrast to murine responses, the addition of Iscomatrix® co-adjuvant was required for optimal antibody responses in the rhesus macaques (Figure 6). Three rhesus macaques injected with a single dose of (NANP)_6_-OMPC/ MAA + Iscomatrix® had GMT 65,020, while the three rhesus macaques immunized with (NANP)_6_-OMPC/MAA had a log lower anti-repeat antibody GMT 6,451. A booster injection of (NANP)_6_-OMPC/MAA + Iscomatrix® on day 70, significantly increased anti-repeat antibody titers 4-fold to GMT 260,080 (corresponding to 481 μg/ml anti-repeat antibody). In marked contrast, in rhesus macaques immunized without Iscomatrix®, the booster injection did not significantly increase antibody titer, with 8,127 GMT post boost as compared to 6,451 GMT post prime.

Persistence of high levels of anti-repeat antibody was also dependent on presence of Iscomatrix® co-adjuvant. Anti-repeat antibody titers in macaques immunized with (NANP)_6_-OMPC/MAA + Iscomatrix® remained high for over 12 months, with a gradual decrease to GMT 25,803 at one year, as compared to GMT 202 in the absence of Iscomatrix®. Reactivity with viable sporozoites expressing *P. falciparum* CS repeats was also maintained in sera of rhesus macaques immunized with (NANP)_6_-OMPC/MAA + Iscomatrix®. CSP titers of ≥1: 512 were obtained in pooled Day 84 sera, decreasing to 1:32 CSP titer on Day 365 (data not shown).

At ~2 years post immunization, rhesus macaques immunized with (NANP)_6_-OMPC/MAA + Iscomatrix® had GMT 12,902 (19 μg/mL anti-repeat antibody). A second booster immunization with (NANP)_6_-OMPC/MAA + Iscomatrix® delivered at this time point elicited strong anamnestic antibody responses. At 1 month post the second boost, antibody levels in the (NANP)_6_-OMPC/MAA + Iscomatrix® immunized macaques reached GMT 206,425 (318 μg/ml anti-repeat antibody), comparable to peak levels observed following first boost. In contrast, at 2 years only low levels were present in rhesus macaques immunized without Iscomatrix® (GMT 160). Following a boost with (NANP)_6_-OMPC/MAA without Iscomatrix® at this time point, no anamnestic response was observed with antibody titers reaching levels observed post first dose, a log lower than levels in the macaques immunized with (NANP)_6_-OMPC/MAA + Iscomatrix®.

### Sporozoite neutralizing antibody *in vitro*

**Figure 7:**
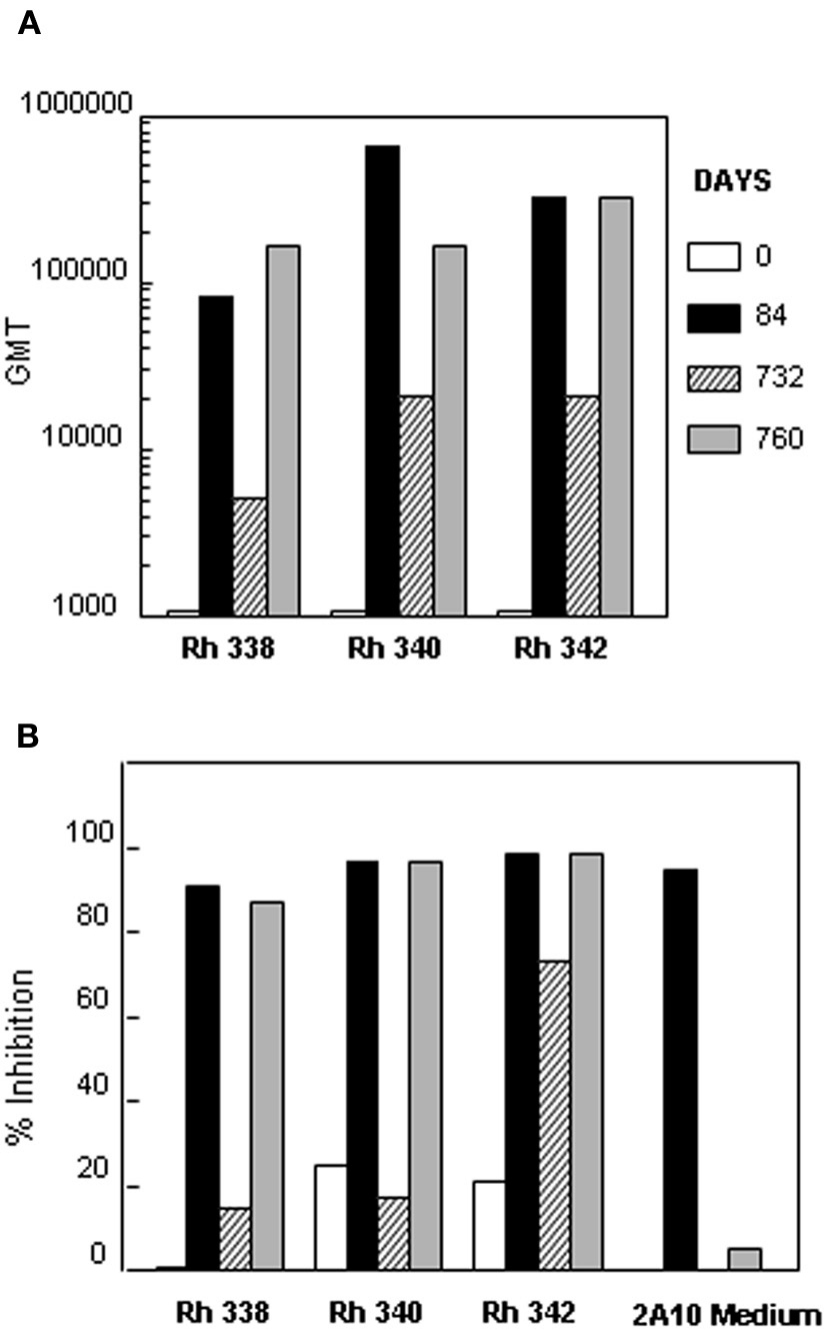
**Sporozoite neutralizing activity in immune sera of rhesus macaques.** Sera was collected from three rhesus macaques at days 0, 84, 732, and 760 post immunization with (NANP)_6_-OMPC/MAA + Iscomatrix®. **(A)** Sera were tested in ELISA with results shown as anti-repeat GMT. **(B)** TSNA was carried out using 1:5 serum dilution with results shown as mean % Inhibition of PfPb sporozoite invasion of HepG2 cells.

The anti-repeat antibodies in the rhesus macaques immunized with (NANP)_6_-OMPC/MAA + Iscomatrix® were functional *in vitro*. Individual sera obtained post the 2nd dose (day 84) had high levels of anti-repeat antibodies in all three rhesus macaques, Rh 338, Rh 340, and Rh 342 (Figure 7A, black bars). Corresponding with the presence of high levels of anti-repeat antibodies, the immune sera of all three rhesus macaques had >90% sporozoite neutralizing activity when tested at 1:5 dilution in the *in vitro* TSNA (Figure 7B, black bars).

Consistent with the ~10-fold decrease in anti-repeat antibodies at day 732, none of the rhesus macaques sera had sporozoite neutralizing activity >90% at this time point (Figure 7A vs. 7B, hatched bars). Importantly, administration of a 2nd booster of (NANP)_6_-OMPC/MAA + Iscomatrix® on day 732 elicited a strong anamnestic antibody response in all three macaques. Serum obtained ~1 month post the second boost (day 760) had high levels of anti-repeat antibody that directly correlated with recovery of sporozoite neutralizing activity (Figure 7A vs. 7B, gray bars). The antibody titers and sporozoite neutralizing activity in day 760 sera was comparable to peak levels observed in day 84 sera following the first boost.

**Figure 8:**
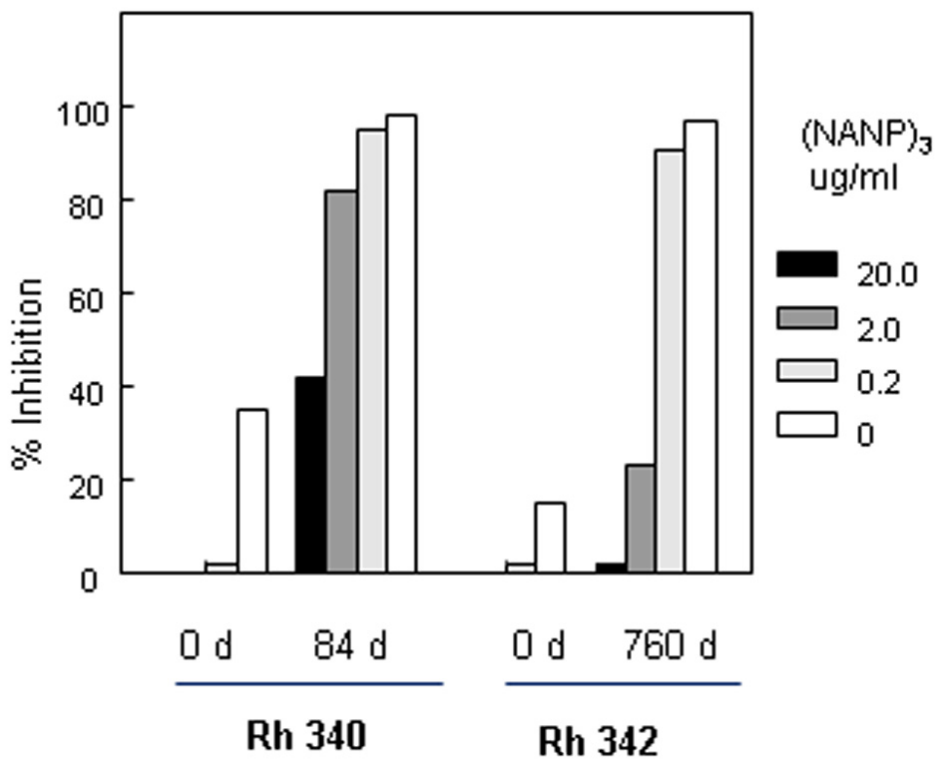
**Peptide competition TSNA.** Rhesus macaque #340 (Rh 340) serum from Days 0 and 84 was tested at 1:10 dilution. Serum from Rhesus macaque #342 (Rh 342) obtained on Days 0 and 760 was tested at 1:20 dilution. Serum was pre-incubated with 10-fold dilutions of (NANP)_3_ competitor peptide prior to addition of PfPb sporozoites and completion of TSNA. Day 0 serum was tested with and without 20 μg/ml of (NANP)_3_ peptide competitor.

Limitations on the number of rhesus macaques available precluded inclusion of an OMPC/MAA only group. In order to rule out non-specific inhibitory effects in the rhesus macaques serum, peptide competition TSNA were carried out to determine if sporozoite neutralizing activity was specific for *P. falciparum* CS repeats. Pre-incubation of rhesus macaques immune sera with various concentrations of (NANP)_3_ repeat peptide was found to block sporozoite neutralizing activity in a dose dependent manner (Figure 8). In Rh 340, immune sera obtained +14 d post first boost (day 84) had 1:10 titer of sporozoite neutralizing activity in the absence of competitor peptide (open bar). Pre-incubation of the immune serum with 20 μg/ml (NANP)_3_ competitor peptide reduced inhibition to levels observed in Day 0 serum (black bar). There was a dose-dependent increase in sporozoite neutralizing activity with lower concentrations of competitor peptide. Rh 340 immune serum incubated with 0.2 μg/ml of (NANP)_3_ competitor peptide gave >90% inhibition of sporozoite invasion of HepG2 cells, similar to sporozoite neutralizing activity in immune serum in the absence of competitor peptide.

Similarly, Rh 342 immune serum obtained on Day 760, ~1 month post the second boost with (NANP)_6_-OMPC/MAA + Iscomatrix®, had high levels of sporozoite neutralizing activity at 1:20 dilution in the absence of competitor peptide (open bar). Pre-incubation of Day 760 immune serum with either 20 μg/ml or 2 μg/ml of (NANP)_3_ competitor peptide reduced inhibition to 5% and 20%, similar to day 0 serum. At low doses of competitor peptide, 0.2 μg/ml, sporozoite neutralizing activity was not blocked and percent inhibition was comparable to Day 760 immune serum without competitor peptide.

## Discussion

In the current study, the (NANP)_6_-OMPC conjugate adsorbed to MAA was found to be highly immunogenic in two inbred strains of mice, Balb/c and C57Bl/6, and in out-bred rhesus macaques. The high levels of anti-repeat antibodies elicited in mice and rhesus macaques correlated with functional antibody that cross-linked surface CS protein in the CSP reaction and inhibited sporozoite invasion of hepatoma cells *in vitro*. These assays demonstrate the ability of antibodies elicited by (NANP)_6_-OMPC immunization to recognize CS repeats on the viable sporozoite and block motility required for invasion of host cells *in vitro* (Cochrane et al., 1976; Stewart et al., 1986).

Although the transgenic *P. berghei* parasites express *P. falciparum* CS repeats, they remain a rodent parasite biologically and thus allow comparison of inhibition *in vitro* with functional sporozoite neutralizing activity *in vivo.* (NANP)_6_-OMPC/MAA immunized mice with high levels of inhibitory antibodies in the *in vitro* assay, had significantly reduced parasite levels in the liver at 48 h post challenge exposure to the bites of PfPb infected mosquitoes. Moreover, the reduced parasite levels in the liver of immunized mice correlated with absence, or delayed, blood stage infection, as measured in Giemsa-stained blood smears. Partial protection in several of the challenged immunized mice was reflected in increased pre-patent periods, indicating that majority but not all infective sporozoites were neutralized.

Anti-repeat antibodies function *in vivo* by immobilizing sporozoites in the skin and preventing entrance into the blood circulation and transit to the liver and/or hindrance of CS interaction with host liver cells required for invasion (Cerami et al., 1992, 1994; Vanderberg and Frevert, 2004). Thus, a key goal of vaccine development is the identification of adjuvant and delivery systems to produce high levels of anti-repeat antibodies. While mice gave optimal responses with alum adsorbed peptide conjugate, Iscomatrix® was required as co-adjuvant for optimal antibody responses in non-human primates. Rhesus macaques immunized with (NANP)_6_-OMPC/MAA + Iscomatrix® had a log higher titer of anti-repeat antibodies compared to rhesus macaques immunized with (NANP)_6_-OMPC/MAA. The antibodies elicited by two doses of (NANP)_6_-OMPC/MAA + Iscomatrix® were long-lived and could be detected for ~2 years post immunization. Encouragingly, a booster immunization at day 732 increased antibody to levels observed post 2nd dose. In contrast, in the absence of Iscomatrix® co-adjuvant, the anti-repeat antibodies were logs lower in magnitude, did not give secondary responses and declined rapidly to near background levels. Similar strong adjuvant requirements were noted in rhesus macaques immunized with an influenza peptide conjugate, M2e-OMPC, formulated in MAA plus QS21, as compared to MAA only (Fan et al., 2004).

Consistent with the murine studies (Figure 4), the presence of high levels of anti-repeat antibody in rhesus macaques immunized with (NANP)_6_-OMPC/MAA + Iscomatrix® correlated with sporozoite neutralizing activity in the TSNA. All three rhesus macaques had neutralizing activity >90% at 1:5 dilution in sera obtained post the first boost, and 2/3 rhesus macaques had neutralizing titers of 1:20. The presence of sporozoite neutralizing activity in serum post the first boost (day 84) and second boost (day 760), directly correlated with the presence of high titers of anti-repeat antibody, with 481 μg/ml and 318 μg/ml, respectively. Inhibition was specific for *P. falciparum* CS repeats, as pre-incubation of rhesus macaques immune serum with (NANP)_3_ competitor peptide led to loss of sporozoite neutralizing activity in TSNA.

Recent clinical studies of CS subunit vaccines have found an association of high anti-repeat antibody titers and resistance to sporozoite challenge, although a threshold level of protective antibody has not been identified. In two recent Phase I/II trials of the CS-based RTS,S vaccine, immunized malaria-naïve volunteers who developed sterile immunity following *P. falciparum* sporozoite challenge had significantly higher concentrations of anti-repeat antibody (114–188 μg/ml) and multifunctional CD4 + T cells when compared to non-protected individuals (30–73 μg/ml) (Kester et al., 2008, 2009). While direct comparison are difficult, the preclinical studies in mice and rhesus macaques of RTS,S in various adjuvant formulations gave levels of anti-repeat antibodies that were lower and less persistent than antibodies elicited by (NANP)_6_-OMPC/MAA ± Iscomatrix®. Mice immunized with RTS,S, in either the AS02 adjuvant (QS21, MPL in oil-in-water emulsion) or AS01 adjuvant (in which liposomes replace the oil-in-water component), developed peak anti-repeat antibody of ~6 × 10^4^ GMT in ELISA using the R32 recombinant protein as antigen (Mettens et al., 2008). In rhesus macaques, two doses of RTS,S, formulated in either the AS01 or AS02 adjuvant, elicited peak anti-repeat GMT ~1.5 × 10^3^, which decreased to near background by 8 weeks post boost. Following a second boost with RTS,S in either adjuvant, GMT rose to peak titers observed post the first boost, returning to near background at 22 weeks.

The current studies demonstrate that the magnitude and persistence of the anti-repeat response in mice and rhesus macaques immunized with two doses of (NANP)_6_-OMPC/MAA ± Iscomatrix® was significantly higher (1 × 10^5^) and more long-lived (~2 years) than those observed following RTS,S immunization. The kinetics, magnitude, and persistence of anti-repeat antibodies in mice and rhesus macaques immunized with (NANP)_6_-OMPC are consistent with results of our previous studies with a *P. falciparum* Transmission Blocking Vaccine (TBV) (Wu et al., 2006). This vaccine, comprised of a recombinant sexual stage protein, Pfs25, conjugated to OMPC and adsorbed to MAA, elicited high levels of functional antibody in mice and rhesus macaques that inhibited oocyst formation in the mosquito midgut. Anti-Pfs25 antibodies persisted for 18 months in rhesus macaques and could be boosted by injection of recombinant Pfs25 protein. Following the second boost, the anamnestic antibody responses reached peak titers similar to those obtained after the first boost, as found also for anti-repeat antibodies in the (NANP)_6_-OMPC/MAA+Iscomatrix® immunized rhesus macaques (Figure 6).

The enhanced immunogenicity of these malaria-OMPC conjugates may reflect unique OMPC interactions with the innate immune system. The vesicular structure of OMPC provides a particle with diameter 100–200 nm to enhance uptake by dendritic cells. OMPC is also a known TLR2 agonist, related to the presence in OMPC of porin proteins from the surface of *N. meningitidis* that has been shown to induce DC activation and maturation (Massari et al., 2002; Latz et al., 2004). In addition, the response to repeat peptide in human volunteers is known to be dose dependent and OMPC has a large capacity for peptide chemical conjugation, with ~3,700 mol/mol for (NANP)_6_-OMPC as compared to the expected lower capacity for (NANP)_3_-TT used in the first clinical trials of a synthetic peptide vaccine (Herrington et al., 1987). Adsorption of the peptide conjugates to alum adjuvant may further increase the immunogenicity of the OMPC based conjugates, by stimulating innate immune responses through NLRP3 inflammasome (Eisenbarth et al., 2008; Li et al., 2008). The combination of enhanced uptake by DC, innate immune signaling through TLR and/or inflammasome, and high density of the malaria epitope, may contribute to strong humoral responses in mice.

In contrast to mice, the (NANP)_6_-OMPC/MAA formulation was not optimal for rhesus macaques. Induction of high levels of long-lived anti-repeat antibody in macaques required inclusion of a co-adjuvant, Iscomatrix®. The (NANP)_6_-OMPC/MAA+ Iscomatrix® formulation elicited titers 2 logs higher than the MAA only formulation, and these antibodies persisted for ~2 years. Iscomatrix® is a particulate adjuvant, comprised of cholesterol, phospholipid, and saponin, which has been shown to be safe in a series of clinical trials of cancer and viral vaccines (Sun et al., 2009; McKenzie et al., 2010). The requirement for stronger adjuvant formulation in rhesus macaques as compared to mice has been found in other studies. In general, a lower immunogenicity of malaria subunit vaccines has been observed in non-human primates. We have found that a stronger oil-based adjuvant was required for optimal immunogenicity of a CS virus-like particle (VLP) vaccine in rhesus macaques as compared to murine studies (Langermans et al., 2005). However, the formulation of VLP in stronger oil-in-water adjuvants was associated with unacceptable reactogenicity, with sterile abscesses developing in the immunized rhesus macaques. No reactogenicity was noted in rhesus macaques immunized with (NANP)_6_-OMPC/MAA + Iscomatrix® in the current studies.

The OMPC carrier has a strong safety record based on its use as carrier in an *H. influenzae* type b (Hib) pediatric vaccine, PedvaxHIB®, which is comprised of *H. influenzae* capsular polysaccharide conjugated to OMPC and adsorbed to MAA. *Hib* vaccine has been delivered to millions of infants 2–71 months old to prevent childhood bacterial meningitis. The well-established safety record of OMPC as carrier protein is particularly important, as the target population for a malaria vaccine is infants <5 years who suffer the majority of the mortality and morbidity caused by *P. falciparum* infection. While PedvaxHIB® vaccination can elicit anti-carrier antibodies that could potentially modulate immune responses to a (NANP)_6_-OMPC vaccine, we found comparable malaria-specific antibody responses induced in mice with and without pre-existing anti-OMPC antibodies. Moreover, the presence of pre-existing anti-OMPC antibodies did not inhibit sporozoite elicited anamnestic anti-repeat antibody responses in (NANP)_6_-OMPC immunized mice, suggesting that parasite-specific memory B cells were also not affected. These studies also suggest that antibody responses induced by (NANP)_6_-OMPC immunization can potentially be boosted by natural exposure to malaria infected mosquitoes in endemic areas.

The studies in mice and rhesus macaques demonstrate that OMPC conjugated to a large malaria recombinant protein (Pfs25H, MW 20 kDa) as well as a low molecular weight (NANP)_6_ peptide can elicit high levels of long-lived functional antibodies that inhibit parasite development *in vitro* and *in vivo*. The combination of both Pfs25 and (NANP)_6_-OMPC conjugates in a vaccine could prevent transmission of parasites both to the mammalian host and to the mosquito vector. Combination vaccines would be particularly effective in malaria control programs, as parasites that evaded the immune responses targeting sporozoites could be prevented from spreading to the mosquito vector. The OMPC carrier would also be amendable to conjugation with other malaria antigens that are targeted by antibody-mediated immunity, e.g., antigens of asexual blood stages that cause clinical disease, to provide multi-antigen vaccines that target all stages of the complex *Plasmodium* life cycle. The current and previous studies in immunized mice of different MHC haplotype and a non-human primate species suggests that OMPC conjugates may lead to new vaccine candidates for control and eventual eradication/elimination of malaria.

### Conflict of interest statement

Investigators at New York University School of Medicine (Elizabeth Nardin, Daniel Carapau and Robert Mitchell) report no conflict of interest. All other authors are current or past (David Kaslow, Elizabeth Ottinger) employees of Merck Research Laboratories.
